# Lipid metabolism alteration contributes to and maintains the properties of cancer stem cells

**DOI:** 10.7150/thno.41388

**Published:** 2020-05-30

**Authors:** Huangcan Li, Zhuoying Feng, Ming-Liang He

**Affiliations:** 1 Department of Biomedical Sciences, City University of Hong Kong, Hong Kong, China.; 2 CityU Shenzhen Research Institute, Nanshan, Shenzhen, China.

**Keywords:** Cancer stem cells, lipid metabolism, signalling pathways, self-renewal

## Abstract

Lipids, the basic components of the cell membrane, execute fundamental roles in almost all the cell activities including cell-cell recognition, signalling transduction and energy supplies. Lipid metabolism is elementary for life sustentation that balances activity between synthesis and degradation. An accumulating amount of data has indicated abnormal lipid metabolism in cancer stem cells (CSCs), and that the alteration of lipid metabolism exerts a great impact on CSCs' properties such as the capability of self-renewal, differentiation, invasion, metastasis, and drug sensitivity and resistance. CSCs' formation and maintenance cannot do without the regulation of fatty acids and cholesterol. In normal cells and embryonic development, fatty acids and cholesterol metabolism are regulated by some important signalling pathways (such as Hedgehog, Notch, Wnt signalling pathways); these signalling pathways also play crucial roles in initiating and/or maintaining CSCs' properties, and such signalling is shown to be commonly modulated by the abnormal lipid metabolism in CSCs; on the other hand, the altered lipid metabolism in turn modifies the cell signalling and generates additional impacts on CSCs. Metabolic rewiring is considered as an ideal hallmark of CSCs, and metabolic alterations would be promising therapeutic targets of CSCs for aggressive tumors. In this review, we summarize the most updated findings of lipid metabolic abnormalities in CSCs and prospect the potential applications of targeting lipid metabolism for anticancer treatment.

## Introduction

**Figure 1:**
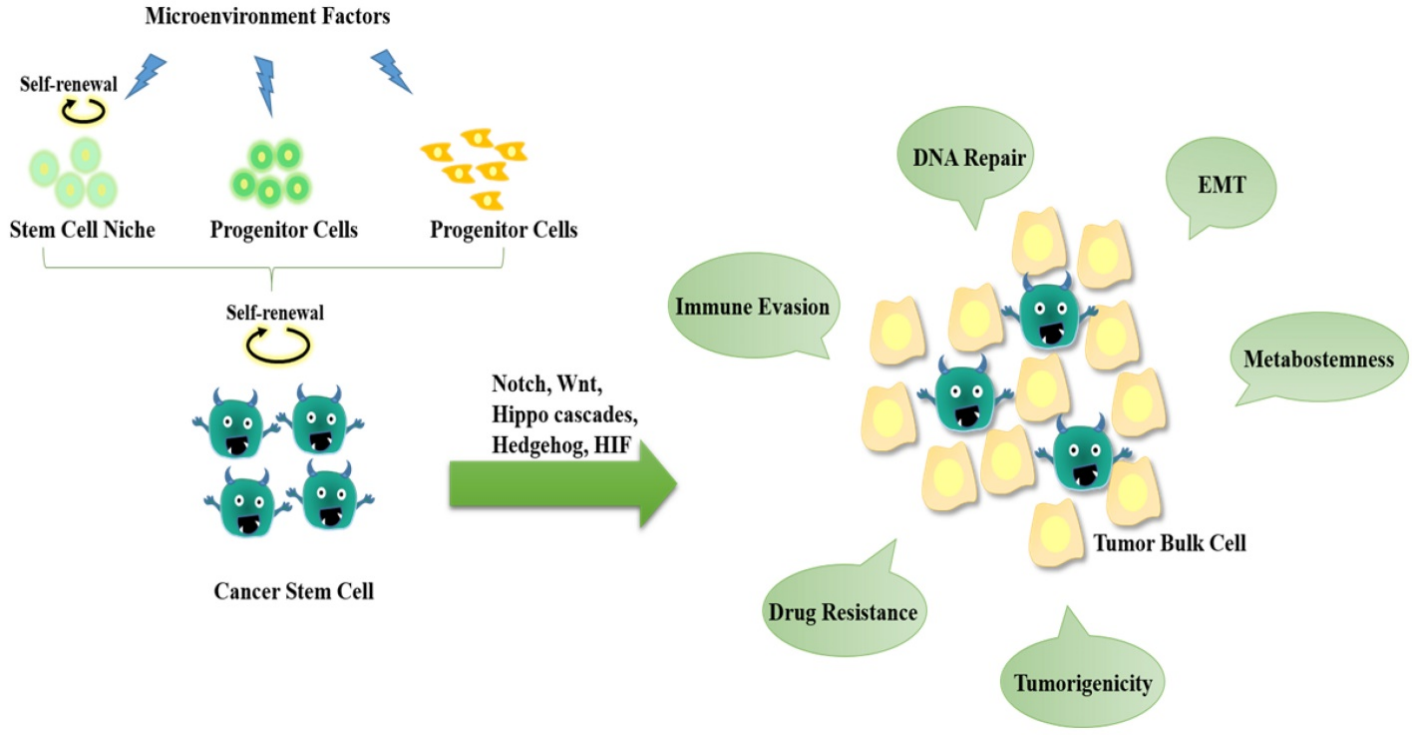
** The hallmarks of cancer stem cells (CSCs).** CSCs may originate from either normal stem cells (including progenitor cells) or transformation of differentiated cells through reprogramming by genetic instability and epigenetic abnormality under long-term stress conditions (e.g., microenvironment factors, hypoxia, virus invasion, etc.). CSCs display a close association with tumor microenvironment. The self-renewal capability of CSCs is significantly enhanced to certain degrees similar to or even stronger than that of the normal pluripotent and multipotent stem cells, but the differentiation capacity is somewhat lost. During the epithelial-mesenchymal transition (EMT) process, cancer cells acquire stem-cell like properties to allow migration and invasion to foreign tissues. CSCs are hierarchical plasticity subpopulations, which are the main causes for tumorigenesis, immune evasion, EMT, tumor progression, metastasis, and drug resistance. These characters of CSCs are regulated by several signalling pathways, such as Notch, Wnt, Hippo cascades, Hedgehog, hypoxia-inducible factor 1 (HIF), etc.

Cancer stem cell (CSC) is a proportion of abnormal cell lineages involved in tumor initiation, progression and metastasis during tumorigenesis (Figure 1), are believed the major cause of drug resistance and recurrence after a period of anticancer chemotherapies. CSCs are similar to or even enhanced self-renewal of the normal pluripotent and multipotent stem cells but lose a certain degree of differentiation capacity 1,2. Two potential origins of CSCs are suggested--either derived from normal stem/progenitor cells through transformation/reprogramming or be transformed from fully differentiated cells caused by genetic instability and epigenetic abnormality during neoplasia pathology 3. Evidence shows that adenomatous polyposis coli (APC) deleted crypt stem cells could induce intestinal microadenomas by activating the Wnt signalling pathway 4. Besides, CSC surface markers, such as CD133^+^, CD44^+^, CD34^+^ CD166^+^, CD24^+^ and ALDH1^+^, also are well known stem cell markers. The study on cell-type plasticity shows that the Wnt-activation in intestinal epithelial cells (IECs) induces non-stem cancer cells' dedifferentiation and crypt stem cell expansion 5. Results from methylation-specific polymerase chain reaction (PCR) experiments reveal that the activated IL-6/JAK2/STAT3 signalling promotes the proliferation of lung cancer stem cells by suppressing p53/p21 expression via DNA hypermethylation 6. During epithelial-mesenchymal transition (EMT), a complicated, multistage process for epithelial cells to enhance motility, weaken cell polarity and degrade the extracellular matrix (ECM) 7, both normal and cancer cells acquire stem-cell like properties to allow migration and invasion to foreign tissues 8.

CSCs have been detected in different types of tumors, including lung cancer 6, breast cancer 9, ovarian cancer 10, pancreatic cancer 11, human acute myeloid leukemia (AML) 12, and glioblastoma (GBM) 13. Regardless of the origin, CSCs are hierarchical plasticity subpopulations driving tumor progression and chemotherapy resistance, thereby hindering tumor prognosis and promoting tumor recurrence. Besides the abnormalities of signalling activations, increasing data have shown that the abnormalities of lipid metabolism exhibit great impacts on CSC properties.

## Abnormal microenvironment and metabolic pattern in tumors and CSCs

CSCs are a group of subpopulation cells in carcinoma. Numerous studies demonstrate that CSCs are responsible for driving tumor growth, epithelial-mesenchymal transition (EMT), metastases and drug resistance. Alternated nutrient consumption between tumor bulk cells and CSCs in tumor microenvironment (TME) is associated with tumor immune evasion and progression. Induced by oncogenes, CSCs facilitate adaptive metabolic changes to sustain increasing energy need for growth and anabolic functions. Similar to stem cells, CSCs exhibit high plasticity in response to the metabolic changes in maintaining self-renewal, proliferation, and survival 14. The metabolic phenotype of CSCs may be heavily decided by microenvironmental conditions. The metabolisms of CSCs are specifically varied, dependent on tumor types and the site of metastasis. Metabolic alternation of CSCs has been proposed as a functional marker and promising therapeutic target.

### Warburg effect

The suffering of cancer cells from abnormal limitations in nutrient supply (such as glucose and oxygen) is referred to as the “Warburg effect” 15. Warburg effect describes a metabolic shift from oxidative phosphorylation (OXPHOS) to glycolysis in pentose phosphate shunt and an accumulation of lactate in exchange for sustained ATP production in TME 16. Emerging evidence has suggested that the glycolytic metabolism of Warburg effect plays a role in stemness and the EMT process 17. R406, a Syk inhibitor for immune thrombocytopenia (ITP), inhibits neurosphere formation and triggers apoptosis in GBM through inducing a metabolic shift from glycolysis to OXPHOS and subsequently producing excessive reactive oxygen species (ROS) in glioma stem cells (GSCs) 18. In the basal-like breast cancer (BLBC) EMT process, Snail-mediated promoter methylation of fructose-1,6-biphosphatase (FBP1) gives rise to enhanced CSC-like properties and tumorigenicity through increasing glucose uptake and macromolecules biosynthesis, as well as inhibiting oxygen consumption through suppressing mitochondrial complex I activity 19. Internal tandem duplication (ITD) mutation in Fms-like tyrosine kinase 3 gene (FLT3/ITD) accounts for approximately 30% of acute myeloid leukemia (AML) cases. FLT3/ITD is extensively present in leukemia stem cells (LSCs) and is proposed to be a primary event in leukemogenesis in possessing CD123 (IL-3RA) stage, and is the main cause of poor prognosis in patients 20. Results from both *in vitro* and *in vivo* studies demonstrate that FLT3/ITD upregulates aerobic glycolysis through activating mitochondrial hexokinase (HK2) in an AKT-dependent manner. Glycolytic inhibitors cause severe ATP depletion and massive cell death in FLT3/ITD positive leukemia cells 21. Recent findings suggest that Warburg effect persist stem cell metabolism in tumors, as a failure of differentiation 13,22. Clinical studies reveal that lower-level uptake of 18F-fluorodeoxyglucose occurs in well-differentiated tumors while higher level uptake happens in the poorly differentiated group. In GBMs, CSCs under nutrient deprivation shift toward the use of pentose phosphate shunt, which promotes CSCs' self-renewal, proliferation and survival 15.

### Oxidative phosphorylation (OXPHOS)

As opposed to differentiated bulk tumor cells that suffer from the “Warburg” effect, CSCs exhibit a distinct metabolic phenotype--being highly glycolytic or OXPHOS dependent. Cancers can be clustered along the differentiation pathways into two groups, utilizing either glycolysis or oxidative phosphorylation. Each group is decided by tumor subtypes, specific phenotype of CSCs, and tumor microenvironment 23. In an inducible pancreatic cancer mouse model, a subpopulation of dormant tumor cells is found to rely on oxidative phosphorylation (OXPHOS) for survival 24. OXPHOS happens in the mitochondria, with the generation of ROS. In gliomaspheres, CSC expansion also depends on OXPHOS in the mitochondrial respiratory chain to produce energy for survival 25. AML employs higher mitochondria oxidative phosphorylation as compared to non-malignant CD34+ hematopoietic progenitor cells 26,27. In an AML xenograft model, the bone marrow stromal cell is deprived of mitochondria through deriving tunnelling nanotubes in the stimulation of superoxide by NOX2. Inhibition of NOX2 interrupts mitochondrial transfer, increases AML apoptosis, and improves AML mouse survival 28. On the contrary, lung CSCs derived from A549 cells display a low quantity of mtDNA, high mitochondrial membrane potential, low oxygen and glucose consumption and a low intracellular concentration of ATP and ROS 29. Similarly, mitophagy, a selective cleansing of mitochondria through autophagy, facilitates the generation and proliferation of liver CSCs by inhibiting p53 expression 30.

### Lipid metabolism

Lipids are typically classified as lipoids (phospholipid, cholesterol and cholesterol ester, etc.) and fats (triglycerides, TG). Lipoids are essential for a variety of cellular functions, including membrane construction, signalling transduction and other biological activities. TG is the main source of cellular energy. Lipid metabolism is elementary for life sustentation that balances synthesis and degradation. As a prerequisite to maintain cell survival, lipid homeostasis is coordinated by integrated systems to quickly respond to metabolic changes. In an energy-deficient or a nutrient exhausted condition, the cell demand for metabolic intermediates for nutrient synthesis and energy production is substantial. Hence, the role of TGs and cholesterol is especially indispensable in cancer and related diseases. Accordingly, disorder or alternation of lipid metabolisms has been linked significantly with pathogenic infection (bacteria, fungi, and virus), lipid-related diseases (hyperlipidemia, lipid storage disease, obesity, etc.) and pathological cancers. Currently, lipid metabolism has been heralded as a novel and significant target for cancer therapy. Emerging evidence has revealed cancer cell alternations in several aspects including membranes formation, lipids synthesis and degradation, and cellular signalling driven by lipids. In the following sections, we focus on the importance and latest findings of fatty acid and cholesterol metabolisms in CSCs, as well as relevant and promising therapeutic targets for cancer therapy.

## The Effects of Lipid Metabolism Alterations in CSCs

Accumulating evidence has shed light on alterations in lipid metabolism and related pathways. Recently, it has been shown that lipids and lipoproteins, either exogenous (or dietary) uptake or endogenous synthesis, have been shown to have a great impact on maintaining CSCs' properties in tumorigenesis. For example, the fatty acid synthase (FASN), a rate-limiting enzyme for de novo lipid synthesis, is consistently found to facilitate in multiple types of CSCs. Furthermore, lipids and cholesterol are increasingly uptaken or generated through hyper-activating the metabolic routes in tumor stem cells. Single-probe mass spectrometry (MS) study has revealed a remarkable metabolic pattern of live CSCs at the single cell level 31. As compared to non-stem counterpart, CSCs generate more active tricarboxylic acid (TCA) and more abundant unsaturated lipids. Previous studies showed that CSCs require more monounsaturated fatty acids (MUFAs) than their non-stem counterparts in ovarian tumors and glioblastoma, suggesting that lipid desaturation may be an ideal biomarker for CSCs 32-35. Furthermore, a comparison of lipidomic profiles between CSCs and non-stem cancer cells suggests that MUFAs affect the formation and stemness of CSCs 32. As the structural components of cellular membranes, the membrane fluidity is highly dependent on the degree of lipid unsaturation. Low membrane fluidity inhibits metastasis and stemness in breast cancers 36. Of note, treating with saturated fatty acids (SFAs) in proportion with glycerophospholipids suppresses hepatocellular carcinogenesis 37. The high proportion of saturated fatty acids attenuates membrane tension and inhibits symmetric division or pluripotent deficiency, indicating the importance of MUFAs in maintaining CSCs 38. The unsaturated lipids regulated by stearoyl-CoA desaturase-1 (SCD1), nuclear factor κB (NF-κB) and aldehyde dehydrogenases 1 A1 (ALDH1A1) significantly promotes the stemness of colorectal CSCs 31. To further clarify this assumption, a study on a series of 577 breast carcinomas shows that the highly elevated ALDH1 level is correlated with poor prognosis 39. Results obtained from both *in vitro* and *in vivo* studies have highlighted the importance of ALDH activity in CSCs' self-renewal and tumorigenicity. Additionally, elevated fatty acid oxidation (FAO) ensures the energy supplies for the extreme environment alternation in CSCs. Hence, carnitine palmitoyl transferase 1 (CPT1), the critical accelerator of FAO, promotes breast cancer stemness and chemoresistance 40. Besides the glycolysis or oxidative phosphorylation, lipids from adipocytes residing in the microenvironment are also used as an energy source in ovarian cancer and prostate cancer 41,42. CSCs require an accelerated FAO to obtain sufficient metabolic intermediates, such as acetyl-CoA and NADH, to satisfy the needs of ATP generation for self-maintenance and proliferation. In hepatocellular carcinoma (HCC) cells and leukemia-initiating cells, FAO is linked to stem-like properties with de novo fatty acid synthesis 43. Leukemia-initiating cells co-opt the adipose tissue niche to create a supportive microenvironment for leukemic growth and chemoresistance 44. Cholesterol is one of the key components in the cell membrane and lipid raft for signalling transduction in pro-oncogenic and anti-apoptotic pathways. Interfering cholesterol biosynthesis may bring large, additional impacts on the cholesterol content in lipid rafts and the signalling transduction for CSCs' proliferation 45,46. Lipid droplets (LDs) are cytoplasmic organelles originating from the endoplasmic reticulum and/or the Golgi apparatus for fatty acids and cholesteryl ester storage. Studies from Groupwise comparisons show that the accumulation of LDs has a close relationship with tumor proliferation and aggression potential 47. In colorectal CSCs, as revealed by Raman spectroscopy imaging, a high level of LDs is a distinctive marker of CSCs. LDs' level also fluctuates with other well-accepted CSC markers such as CD133, activated Wnt pathway, etc. 48. Furthermore, a statistical analysis of the overall lipid droplets from cancer cells has been considered as an ideal marker of tumor aggressiveness 49.

### Fatty Acids (FAs) homeostasis

Fatty acid (FA) metabolism is the core of lipid status harmonization, which maintains the energy and supplies for the living of organisms. FA synthesis produces the fundamental component of various lipids for cell membrane construction, signal transduction, energy storage, and biological functions. FA catabolic pathway generates energy via FA degradation conducted by FA oxidation (FAO), or commonly known as β-oxidation.

**Figure 2:**
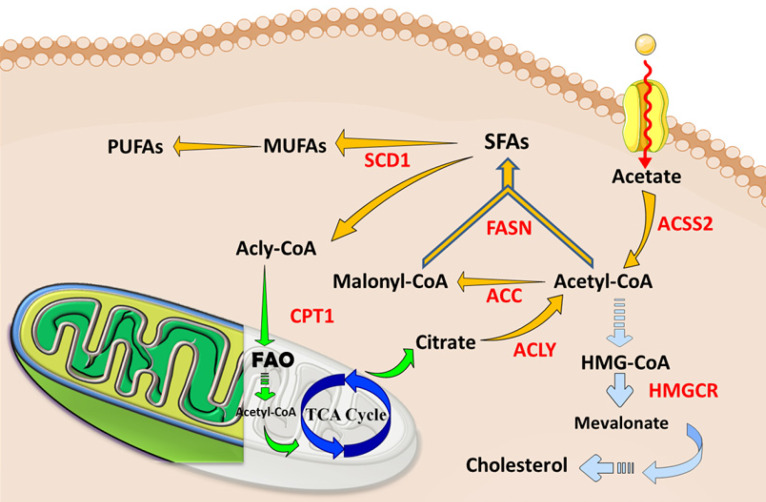
** The abnormal lipid metabolisms and current therapeutic targets in tumors and CSCs.** Cancer cells, especially CSCs, increase lipids catabolic/anabolic activities, such as FA synthesis (Yellow arrows), FA oxidation (Green arrows), and cholesterol synthesis (Blue arrows). The ectopic lipid metabolisms facilitate the pluripotency, self-renewal, proliferation and formation of CSCs. Currently, key enzymes (Red letters) dominating lipid metabolisms have been considered as ideal therapeutic targets or prognosis for cancers. Abbreviation: ACC, acetyl-CoA carboxylase; ACLY, ATP-citrate lyase; ACSS2, acyl-CoA synthetase short-chain family member 2; CPT1, carnitine palmitoyl-transferase 1; FA, fatty acid; FAO, fatty acid oxidation; FASN, fatty acid synthase; SCD1, stearoyl-CoA desaturase1; TCA cycle, tricarboxylic acid cycle; MUFA, monounsaturated FA; SFA, saturated FA; PUFA, polyunsaturated FA. The figure was produced using Servier Medical Art (http://www.servier.com).

In the last years, the importance of lipid metabolism in cancer cells has been repeatedly emphasized, and a series of significant advances have been made to provide useful reference indicators and directions for cancer therapy 50,51. Tumor cells proliferate rapidly while angiogenesis becomes abnormal, thus cancer cells are under hypoxic, hyper-oxidative, acidic and malnutrition conditions. CSCs alter their basic metabolisms to encounter those unfavorable microenvironments. Lipid metabolism presents a massive and complex network of flexible pathways, feedback loops and cross talks that maintains the metabolic requirement for cancer cells. FA homeostasis and balance of FA synthesis, storage, and degradation control the core node of the framework. FA synthesis generates various metabolic intermediates that are fed to anabolic metabolisms for cellular membrane maintenance or signal transduction in inducing oncogenic cascades, resulting in malignancy, chemoresistance and cancer stemness. As a supplement, FAO retrieves acetyl CoA to initiate FA synthesis, indicating that FA synthesis and FAO are mutually complemented. More distinctive than glycolysis contribution in CSCs, lipid metabolism may contribute to CSCs in varied aspects. A recent study shows that fatty acids metabolisms, both FA synthesis and FAO, contribute to the pluripotency and reprogramming of embryonic and somatic stem cells 38,52,53. Compared to the non-stem counterparts, CSCs reduce the utilization of glycolysis but maintain a sufficient amount of ATP generation, indicating the significance of alteration in lipid metabolism 54. Distinctive lipidomics changes have been demonstrated to vary in CSCs and bulk cancer cells in glioblastoma multiform 32. Moreover, the intermediates generated by glycolysis are fed to FA synthesis for CSCs self-renewal 55. Indeed, fatty acid homeostasis, or the balance of catabolic/anabolic state, keeps the grip of pluripotency, self-renewal, proliferation and formation of the CSCs (Figure 2).

#### FA synthesis promotes CSCs

Limited to the absorption and consumption of dietary lipids, cancer cells urgently require FA synthesis to meet energy needs and structural construction. Overall, key players in FA synthesis, such as ATP-citrate lyase (ACLY), acetyl-CoA carboxylase (ACC) and fatty acid synthase (FASN), are elevated in cancer cells. These enzymes are emerging as the hallmark of cancer and even ideal markers for cancer stemness 51,56. Unlike their non-stem counterpart, CSCs may absorb glycolytic metabolic intermediates for lipid biosynthesis to improve self-renewability under the Warburg effect 57. By measuring the ^14^C-glucose and ^14^C-acetate incorporation as the carbon source for *de novo* lipogenesis, studies show GSC requires more lipogenesis than bulk cancer cells in glioblastoma 58. Emerging evidence has emphasized the impact of fatty acid synthesis deficiency in multiple carcinogenesis and cancer stemness, recognizing the inevitable role of *de novo* fatty acid synthesis in CSC self-renewal and survival 59. Here, we mainly compare each key player for its role in constituting fatty acid synthesis and further discuss the potential therapeutic strategies in eliminating CSCs via the anti-lipogenesis method.

##### ATP citrate lyase (ACLY)

ACLY catalyzes the conversion of citrate into acetyl CoA in the cytoplasm, which is the significant building block of fatty acid and cholesterol synthesis. Elevated expression level and activation of ACLY have been broadly reported in multiple tumors. Elevated ACLY activity positively enhances malignant phenotypes and poorer prognosis 38,60,61. On the contrary, inhibition of ACLY suppresses tumor growth and EMT 62,63. ACLY is also indicated as a fundamental factor of cancer stemness. Inhibition of ACLY by siRNAs or chemical inhibitors significantly impairs the growth of CSCs derived from human non-small cell lung carcinoma or breast cancer 64-66. ACLY inhibition decreases the proliferation of lung CSCs activated by Ras or by epidermal growth factor receptor (EGFR) mutations. ACLY knockdown significantly reduces the CSC population in breast cancer cells 62,66. Snail, a crucial transcription factor for EMT induction, is a potential target of ACLY in the process of Ras-induced cancer stemness. The phosphorylation of ACLY at serine 454 by phosphatidylinositol 3‑kinase (PI3K)/protein kinase B (AKT) pathway is up-regulated with the stage, the tumor differentiation grade, and poor prognosis in non-small cell lung cancer 67. Phosphorylated forms are suggested to profoundly increase the effect of ACLY in CSCs.

##### Acetyl-CoA carboxylase (ACC)

ACC, which carboxylates acetyl-CoA into malonyl-CoA, exhibits up-regulation in the breast, gastric, and lung cancers 68-70. Furthermore, the distinctive elevation of ACC and FASN in iPSC emphasizes the importance of lipogenesis in stemness and beacons potential therapeutic utilization in CSCs. Cytosolic ACC inhibition mediated by phosphorylation at serine 80 has been considered as a necessary feature for metastasis and invading behaviour in breast and lung cancers, and this concept may be universal in other types of cancers 70. A study on ACC function in breast cancer indicates an unexpected enzymatic feature, in that the regulation of ACC in metastasis and tumor recurrence depends on the accumulation of acetyl-CoA and protein acetylation instead of its native duty in fatty acids synthesis 62. Wnt/β-catenin signalling also participates in the regulation of ACC in CSCs, because silence of β-catenin induces ACC expression 71.

##### Fatty acid synthase (FASN)

Fatty acid synthase (FASN), the crucial enzyme for the *de novo* lipogenesis, also plays an important role in maintaining cancer stemness. Elevated FASN levels have been reported in a variety of cancers, including liver, prostate, breast, ovarian, endometrial and pancreatic cancers 59. The increase of FASN is highly correlated with poor prognosis and disease recurrence 72. Notably, FASN is overexpressed in iPSCs, while FASN deficiency impairs the reprogramming ability of iPSCs 73. Similarly, adult neural stem and progenitor cells (NSPCs) show a strong dependence on FASN. Inhibition of FASN-mediated lipogenesis decreases NSPC proliferation 74. FASN expression level is regulated by β-catenin 75, and associated with the level of stemness markers (SOX2, CD133 and Nestin) in GSCs 58. In certain cases, FASN levels seems to be positively correlated with ACC expression in CSCs. FASN is suggested to be a more vulnerable target in CSCs than in the bulk cancer cells.

##### Stearoyl-CoA desaturase (SCD)

Several studies also revealed how signalling pathways to promote CSCs via regulation of unsaturated fatty acids. Importantly, NF-κB, the main regulator of tumors and CSCs, directly regulates the expression and activation of lipid desaturases, whereas the abrogation of lipogenesis through desaturases inhibition inactivates AKT/ERK-mediated NF-κB signalling 35,76. Meanwhile, the level of SCD-dependent MUFAs also directly regulates CSCs through Wnt/β-catenin pathway, one of the most significant signalling both in stem cells and in CSCs 77,78. Hippo pathway regulated by YES-associated protein (YAP) and tafazzin (TAZ) promotes embryonic and somatic stem cell renewal and differentiation 79. Interestingly, the activation of SCD1 positively regulates the stabilization and nuclear localization of YAP/TAZ, indicating a significant impact on cancer stemness and the chemotherapy resistance in lung cancer stem cells 80.

In humans, SCDs have two isoforms, SCD1 and SCD5. SCD1 is the major enzyme catalysing desaturation in all tissues while SCD5 mainly expresses in the pancreas and brain 81. Consistent with the performance of MUFAs in CSCs, the increased expression level of SCD1 in the lung, ovarian, breast, and glioblastoma cancer stem cells further emphasizes the importance of MUFAs, speculating a significant role of SCD1 for lipid component regulation in CSCs 80,82-84. Additionally, SCD1 expression level also increases and corresponds with the maintenance of some stem cells, such as bone marrow mesenchymal stem cells, pluripotent stem cells and hair stem cells 85-87. It is found that SCD1 also regulates Wnt signalling in CSCs 75,88. Correspondingly, SCD1 inhibition preferentially diminishes CSCs population in the lung, brain, ovarian, lymphatic and colon cancers; while the functional failure is rescued by the MUFAs, such as oleic acid 32,35,80,82,89,90. However, SCD5 expression and activity may not be prominent in most cancer cells. Intriguingly, the SCD5 expression level is reduced in melanoma; while restoration of SCD5 suppresses the formation of malignant melanoma through a reversed EMT-like process and induction of cancer cell differentiation 91.

#### FAO enhances CSCs

FAO mediated energy generation is particularly critical to cancer cell survival and metastasis, especially to non-glycolytic prostate adenocarcinoma and diffuse large B-cell lymphoma 92,93. Elevated FAO helps cancer cell survival in nutrient deficiency and anoxic microenvironments 94. Ectopic activation of FAO maintains CSCs under conditions of glycolytic deficiency 95,96. FAO is also found to dominate the stemness in mesenchymal stem cells (MSCs) isolated from the advanced stage of gastric cancer (GC), indicating an effective target for reducing chemoresistance 97. Consistently, inhibition of FAO by perhexiline impairs cancer stem cell self-renewal and increases the sensitivity of breast CSCs to chemotherapy 98. Increased FAO has also been speculated to interact with Src oncoprotein and participate in the generation of triple-negative breast cancer stem cells 99. Emerging evidence has revealed the mechanisms behind mitochondrial FAO's contribution to CSCs. ROS reduction caused by FAO impairs stem cells 95, explaining the efficient therapeutic effect through redox defence blockage in CSCs 100. Additionally, mitochondrial FAO contributes to cellular activity and pluripotency in haematopoietic stem cells 101 and adult neural stem cells 102. Therefore, inhibition of FAO exacerbates the symmetric differentiation of adult neural stem cells at the expense of self-renewal abilities 103. On the other hand, elevated peroxisome FAO benefits Tie2+ hematopoietic stem cell proliferation by activation of mitochondrial clearance 101. Though no direct evidence pinpoints the influence of FAO on Notch signalling, Notch1 coordinates FAO for the regulation of lipid accumulation in the liver 104 and redox homeostasis in quiescent endothelial cells 105. FAO and its functions in lipid accumulation provide the grounds for CSCs' for survival under nutrition, environment and energy stress.

### Cholesterol homeostasis

Cholesterol homeostasis mainly relies on two mechanisms 106. On one hand, Cholesterol levels can be upregulated by synthesizing *de novo* from acetyl CoA provided by glycolysis, glutamine metabolism, TCA cycle or exogenous uptake by low density lipoprotein receptors (LDLRs), as well as the so-called reverse cholesterol transport (RCT), allowing peripheral cholesterol to be returned to the liver in low density lipoprotein (LDL) 107. The process of cholesterol synthesis is mediated by the mevalonate (MVA) pathway 108. On the other hand, cholesterol levels can be negatively regulated through the inhibition of the MVA pathway or the activation of liver X receptors (LXRs). The MVA pathway can be reduced through proteolytic processing or nuclear import of sterol regulatory element binding proteins (SREBP2), while LXRs can be activated through cholesterol conversion to oxysterols 109. The activation of LXRs/PPAR pathway, in turn, activates the transcription of the E3 ubiquitin ligase IDOL, which ubiquitinates LDLR and upregulates the cholesterol efflux pump ABCA1 and ABCG1 110. Retrospective and experiment data show that both circulating LDL-cholesterol and elevated dietary cholesterol are associated with a poor progression free survival time (PFS), while statins show protective effects in ovarian cancer 111,112, non-small cell lung cancer 113, breast cancer 114, pancreatic tumour 115, colorectal cancer 116, and so on. SREBP2 is found to promote stem cell-like properties and metastasis by transcriptional activation of c-Myc in prostate cancer 117.

Cholesterol is the major sterol in mammals and is especially critical for cell growth and function. Besides acting as a precursor for sterol hormones, bile acids, vitamin D and oxysterols, as well as major components for membrane reinforcement, cholesterol regulates cell signalling via lipid rafts. Various of proteins have been discovered in lipid rafts, such as caveolins 118, src family kinases 119, MAP kinase (MAPK), protein kinase C, EGFRs 120, flotillins, low molecular weight heterotrimeric G proteins 121,122, platelet-derived growth factor (PDGF) receptors 123, endothelin receptors and so on. Proteins are selectively included or excluded from the membrane microdomains, which serve as rafts for the transportation of specific domains or relay stations for transducing intracellular signalling 124. In most cases, lipid rafts act as signalling platforms that combine the necessary components, manage their interactions and transduce pathway signalling 125. Different lipid rafts could also be united as complementary components of a signalling pathway. In turn, pathway signalling could be regulated by lipid rafts compartmentalization through locational and physical separation of proteins 121,126,127. For example, CD133+ pancreatic tumor initiating cells (TIC) shows high-level expression of MAPK with high cholesterol content. Furthermore, the study also shows that CD133 is localized in the lipid rafts. Disruption of lipid rafts decreases metastatic potential and chemoresistance in CD133+ cells but does not affect the CD133- cells, resulting in deregulation of focal adhesion kinase (FAK)-signalling 128.

#### Cholesterol facilitates CSCs tumorigenesis

Cancer was first linked to nutrition by epidemiological studies, demonstrating that environmental factors such as diet and nutrition are important in carcinogenesis 129. Caloric intake, types and amount of fats, proteins, amino acids, vitamins, minerals, fibers, and other dietary constituents have been studied regarding their influence on tumorigenesis. In particular, increased cholesterogenesis is associated with tumorigenesis through activation of tissue growth and loss in feedback control. Early laboratory studies elicited the role of cholesterol in cancer development and progression 45. Lipoproteins are capable of stimulating growth and metastasis of cancer cells* in vivo* and *in vitro* 49,130,131. Accelerated evidence also shows that cholesterol and FA metabolism are the hallmarks of cancer, contributing to malignant transformation due to the obligatory requirement of cholesterol for cell membrane functions. In mammosphere models generated from breast patient-derived xenograft (PDX) tumors, GO enrichment analysis identifies cholesterol biosynthesis to be the most significantly activated process 132. Furthermore, the high levels of cholesterol biosynthesis-related proteins are associated with short relapse-free survival in basal-like breast cancer patients 33. In a cohort of 615 basal-like breast cancer patients, except for DHCR7 or LSS, all cholesterol synthesis-associated proteins show a significant correlation between higher level of gene expression and shorter relapse-free survival 9. In another analysis, enzymes of the MVA metabolic pathway are overexpressed in breast cancer stem cell tumorspheres as compared to cognate adherent cells. A small-molecule inhibitor of the geranylgeranyl transferase (GGTI) reduces the breast CSC population both *in vitro* and *in vivo* 133. Phospholipid remodeling enzyme lysophosphatidylcholine acetyltransferase 3 (LPCAT3), which incorporates polyunsaturated fatty acids into phospholipids, is a crucial determinant of membrane lipid composition. Lack of LPCAT3 in intestinal stem cells leads to an excess of cholesterol production in response to changes in phospholipid composition, resulting in intestinal stem cell hyperproliferation 46. In multiple brain tumor-initiating cells, several genes in MVA pathway, including HMGCR, PMVK, MVK, MVD, IDI1 and FDPS, are highly expressed 134.

Bile acids and oxysterol are two chemical by-products in the MVA pathway. They act as ligands for a family of nuclear receptors (including FXR, VDR, LXR and PXR) and G-protein-coupled receptors 135 136. As reported in colorectal cancer, the high-fat diet and dysregulated WNT signalling pathway alter bile acids profiles, activate FXR, and drive malignant transformations in Lgr5^+^ subpopulation CSCs 137. Oxysterols are a group of Janus molecules result from enzymatic oxidation of cholesterol's side chain, can induce both the early inflammatory reaction against cancer expansion or apoptosis and sustain a complex survival signalling pathway in favor of the neoplastic process 138.

#### Abnormal cholesterol metabolism in CSCs

Cancer cells adapt to maintain high intracellular cholesterol similar to the normal homeostasis including accelerated endogenous production of cholesterol and fatty acids regulated by the SREBPs, or by reducing cholesterol efflux trough ABC class A transporters such as ABCA1, or by increasing the uptake of LDL.

Loss of phospholipid-remodelling enzyme Lpcat3 or activation of SREBP-2 in APC-defect mice markedly promotes intestinal tumor formation by modulating intestinal stem cell homeostasis and tumorigenesis 139. HMG-CoAR is the rate-limiting enzyme in the MVA pathway and the popular cholesterol synthesis lowering agents 131. Statins, the inhibitors for HMG-CoAR, reduces tumor-like sphere formation and exhibits high therapeutic indices 140. This study indicates that HMGCR may be a predictive marker for statin therapy 141. Overexpression of ABCA1 contributes to drug resistant in subpopulations of CSCs (EpCAM^+^ CD45^+^ CD133^+^ and CD117^+^ CD44^+^) in epithelial ovarian carcinoma patients 142. The scavenger receptor, class B type 1 (SRB1), is a multiligand membrane receptor protein that functions as high-density lipoprotein (HDL) influx receptor of HDL-derived cholesteryl esters into cells and tissues 136. SRB1 also facilitates the efflux of cholesterol from peripheral tissues back to the liver 143. SRB1 may be responsible for an increased cholesterol uptake by the tumor and indirectly regulate tumor development. In the western diet mice models, SRB1 is highly expressed in the transformed prostatic epithelial cells and is responsible for an increased cholesterol uptake sustaining tumor development 144. The higher affinity of LDL in tumor cells is detected, the increased activity of HMG-CoAR is observed 145,146. LDL macromolecule has been developed as a specific delivery for cytotoxic drugs or radio nucleotides 147, specifically in CML patients where the poor prognosis is linked to low plasma lipid concentrations 148.

#### Signalling modulated by cholesterol in CSCs

MVA pathway is highly conserved in all the eukaryotes. Other intermediate products, such as farnesyl pyrophosphate, squalene, isoprenoids, lanosterol, bile acids, steroid hormones and vitamin D, participate in cellular physiological functions. Deregulation of cholesterol homeostasis has been proven a powerful way to suppress oncogenic receptors' signalling for inhibiting tumor growth. Cholesterol mainly promotes cancer signalling in two ways. Cholesterol itself as well as chemical by-products from the mevalonate cascade exerts a tumor progression effect. On the other hand, as a critically important component in lipid rafts, cholesterol can affect receptor affinity and the activity of proteins via sterol-sensing domains, including EGFR family, G-protein coupled receptors (GPCRs), PI3K and AKT 146.

In the Hh signalling pathway, lipid modification is crucial for its biological function. Cholesterol covalently bound to the cleaved C-terminal end Gly257 of the N-peptide in Shh translation is an essential step for Shh maturation. The genetic defects in cholesterol biosynthesis can cause a subset of genetically determined anatomical defects, termed holoprosencephaly (HPE), which results from Shh signalling blockage in embryonic development 149. Besides, the GPCR-like protein Smoothened (SMO), as an orphan GPCR that transmits the Hh signal, can be directly activated by cholesterol. Cholesterol together with some other oxysterols, such as 20(S)-hydroxylcholesterol, 22(S)-hydroxylcholesterol and 7-keto-25-hydroxylcholesterol, is identified as potent activators of SMO through its extracellular cysteine-rich domain (CRD) 150,151. Sterol depletion reduces SMO accumulation on the primary cilium. However, neither the Notch signalling nor the Hippo cascades could directly interact with cholesterol. Instead, Notch signalling can be modulated by the lipid composition of the cell membrane, in addition to the O-glycosylation of the receptor 152. A high-content with high-throughput screening on FDA -approved drug library shows the strongest YAP/TAZ inhibitory effect in all of the five statins present in the library 152. MVA pathway activity, mainly the geranylgeranyl pyrophosphate (GGPP), is required to sustain the YAP/TAZ gene expression program. Only the geranylgeranyl transferase inhibitor GGTI-298 is shown to rescue the effect of statins on YAP/TAZ localization, while the squalene synthase inhibitor (YM-53601) or farnesyl transferase inhibitor (FTI-227) fails to converse the effect. GGPP, crucial for the enzymatic activity of Rho small GTPases located in the plasma membrane, reduces the inhibitory phosphorylation of YAP/TAZ and sustains YAP/TAZ nuclear accumulation 153. Inhibitor of the geranylgeranyl transferase effectively reduces the growth of breast CSCs both *in vitro* and *in vivo* 133. CSCs' sphere-forming is suppressed through inhibiting RhoA and increasing P27^kip1^ accumulation that finally leads to inhibition of RB phosphorylation and cell cycle arrest in cancer cells. These findings are considered as promising perspectives for target-based cancer therapy 131,133.

Cholesterol metabolism has also been explored its important roles in immunity. Current studies indicate a significant connection between cholesterol metabolism and immunotherapy resistance. The plasticity and ability of CSCs enable them to interact with TME, to modulate and shape immune responses, thus resulting in immune impairment and tumor recurrence 154,155. Among the numerous immune cells, T cells (especially CD8^+^ T cells) have exerted an important function in IFN-γ and TNF (tumor necrosis factor) signalling 156. However, the activity of CD8^+^ T cells is suppressed in tumor microenvironments. Nevertheless, the increased cholesterol level decreases the T cell antigen receptor (TCR) activity and nanoclusters, which are critical for antigens recognition and binding to major histocompatibility complex molecules (MHC) on other cells 157.

## Important signalling pathways involved in lipid metabolism of CSCs

**Figure 3:**
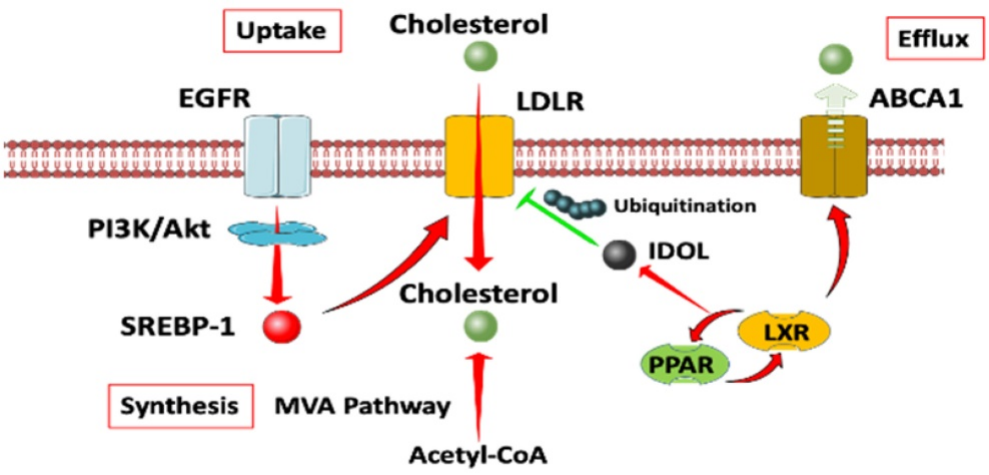
** A diagram of cholesterol homeostasis.** Cholesterol uptake is mediated by LDLR through EGFR dependent pathway. Cholesterol synthesis goes through MVA pathway. LXR plays a crucial role in both the negative control of cholesterol uptake and regulation of cholesterol efflux. Abbreviation: EGFR, epidermal growth factor receptor; LDLR, low density lipoprotein receptors; ABCA1, ATP-binding cassette transporter 1; PI3K/Akt, phosphatidylinositol 3‑kinase/protein kinase B; SREBP-1, sterol regulatory element binding proteins; MVA, mevalonate; IDOL, inducible degrader of the low-density lipoprotein receptor; PPAR/LXR, lipid-activated transcription factors /liver X receptors.

In stem cells, several important signalling pathways involved in lipid metabolism participate in controlling self-renewal, embryonic development and lineage specification. Since CSCs can be derived from stem cells through genetic mutations and epigenetic alteration, it is highly likely that these pathways are hijacked to maintain the unrestrained proliferation, invasion and drug resistance 158. In CSCs, a series of pathways involved in lipid metabolism maintains the undifferentiating state, guide the lineage progeny and sustain their survival as well as proliferation (Figure 3), including Notch signalling 159-161, Hippo cascades, Hedgehog (Hh) signalling, and Wnt signalling 9,153,154.

### Notch signalling pathway

Notch signalling pathway is one of the most conserved signalling pathways activated in embryonic vasculature development 160. In Drosophila, Notch signalling is sensitive to environmental sterol levels. The expression level of Notch signalling is modulated by dietary cholesterol, resulting in intestinal cell differentiation from stemness status 162. In cancer cells, Notch pathway plays a critical role in angiogenesis, EMT and CSCs proliferation 159,163. The low-sterol diet restricts the growth of enteroendocrine tumors by decrease of Notch responses 162. Interestingly, Notch1 controls FAO to achieve intermediate lipid homeostasis and redox homeostasis in CSCs 104,105. Exogenous lipids are demonstrated to positively regulate Notch signalling. In human beings, Notch signalling can be modulated by the lipid composition of the cell membrane 153.

### Hippo signalling

Emerging evidence shows the intimated association and the importance of hippo-YAP/TAZ signalling in lipid metabolisms in regulating cancer stemness 80,164. Hippo signalling mainly functions through phosphorylating the plasma membrane receptor MST1/2, followed by phosphorylation and activation of MOB1A/B and LATS1/2, causing sequester and proteasomal degradation of YAP and TAZ by the 14-3-3 phosphopeptide binding proteins. In CSCs, the activation of YAP or TAZ sustains self-renewal and tumor-initiation capacities 165, promotes cell pluripotency 166 and drug resistance 167, and is highly related to EMT process 167-171. The main MUFAs regulator SCD1 contributes to cancer stemness through the regulating YAP/TAZ in both expression and nuclear localization 80. As an intermediate controlled by the MVA pathway, GGPP is a sufficient factor for the stabilization of YAP/TAZ 153.

### Hedgehog (Hh) signalling

When the three Hh ligands, Sonic (Shh), Indian (Ihh), and Desert (Dhh), binding and inhibiting the Patched 1 (Ptch1) and/or Patched 2 (Ptch2) receptors, the repression of Smoothened (SMO) is relieved, followed by activation of the GLI transcription factors (GLI-TFs) GLI1, GLI2, and GLI3 172. The Hh signalling cascade leads to transcription of Hh-targeted genes, such as Cyclin D1 and c-Myc 173. Hh ligands are found to activate in colon cancer, as well as other solid tumors. The enhanced Hh signalling accelerates the progression of advanced neoplasms 174. Hh signalling also plays a crucial role in CSCs. Hh signalling modulates the postnatal mammary stem cells (MASCs) proliferation and generates the complex ductal structure of the adult mammary gland 175. In breast cancer EMT programs, primary ciliogenesis activates the Hh signalling that enables the stemness and the tumor-forming capacity of stem cell-like tumor-initiating cells 176. Lipid metabolism is also known to regulate hedgehog signalling and its ligand properties 177. Cholesterol is crucial for Shh maturation and can directly activate the SMO receptor in Hh signalling 149,150. The genetic defects in cholesterol biosynthesis causes a subset of anatomical defect holoprosencephaly (HPE), resulting from Shh signalling blockage in embryonic development 149. Recently, SMO inhibitors and GLI inhibitors are used to target the Hh signalling pathway in clinical trials 178,179.

### The Wnt signalling pathway

In the canonical Wnt pathway, Wnt ligands bind to the transmembrane receptor Frizzled (Fzd) family, leading to activating Dishevelled (Dvl) and then triggering the stabilization and accumulation of nuclear β-catenin transcriptional activity, in cooperation with T-cell factor (TCF)/lymphoid enhancer factor (LEF) family. Other co-receptors, such as low-density lipoprotein-related protein (LRP5/6) or tyrosine kinase receptors (PTK7, ROR, RYK), may also act as cofactors in the canonical Wnt pathway. The non-canonical signalling (β-catenin-independent pathway) consists of the Wnt/Ca^2+^ pathway and the planar cell polarity (PCP) pathway. The Wnt signalling pathway plays a highly evolutionarily conserved role in embryonic proliferative tissue development (such as hematopoietic system, skin and intestine) for body axis patterning, cell fate specification, cell proliferation and migration 180. In tumorigenesis, the Wnt signalling promotes tumor migration and invasion by upregulating genes involved in cell adhesion, including Eph/Ephrins, E-cadherin and MMPs 181. However, in the hypoxic GBM patient-derived cell lines, TCF1 and HIF-1α together inhibit the expression of stemness markers Nestin and CD133 through activation of Wnt signalling that reduces the GBM stem cell frequency and strongly increases acquisition of neuronal traits​ 182,183. In squamous cell carcinoma, depletion of β-catenin halts tumor progression, suggesting its roles in the maintenance of cutaneous CSCs-like properties 181. The Wnt signalling also cooperates with lipogenesis in cancer cells 75. The Wnt/β-catenin signalling significantly modulates *de novo* lipogenesis, which is characterized by a significantly increased expression of ACC, FASN, and sterol regulatory element binding protein-1c (SREBP1-c) in breast cancer cells 71. FAO has been identified as an enhancer for β-catenin expression in HCC 184. Importantly, SCDs have been considered as the key factor in regulating Wnt signalling in CSCs 75,88. In colorectal cancer, a high-fat diet and dysregulated WNT signalling pathway alter bile acids profiles, activate FXR, and drive malignant transformation in Lgr5^+^ subpopulation CSCs, which promote an adenoma-to- adenocarcinoma progression 137.

### Other signalling pathways involved in lipid metabolism in CSCs

Other pathways, such as EGFR signalling 185,186, signal transducer and activator of transcription (STAT) signalling, PI3K/PTEN/Akt/mTORC1/GSK-3 pathway 186, ephrins and bone morphogenetic proteins (BMPs) signalling 187, and NF-κB signalling, have been studied in CSCs for many years. Pharmacological agonists/inhibitors targeting such pathways are in clinical trials 188. For example, the member of STAT family ultimately regulates tumor stem cell self-renewal, differentiation, and apoptosis 189. Activation of JAK/STAT3 signalling promotes CPT1 expression, resulting in the reinforcement of cancer stemness and chemoresistance in breast cancer 98.

## Targeting lipid metabolism for anticancer treatment

The importance of lipid metabolism in CSCs has been continuously studied and emphasized that the inhibitors targeting each participant in FAS, FAO and cholesterol metabolisms are widely tested in cancer treatment and chemotherapy assistance.

### Targets on FAS

Promisingly, therapeutic targets on ACC and FASN achieve reliable results in elimination of CSCs or cancer therapy. ACC inhibitor, such as **Soraphen A**, has been considered as a treatment option by targeting lipogenesis in breast CSCs 190. Additionally, chemical compounds with the same binding site as Soraphen A can inhibit the growth and proliferation in non-small cell lung cancer (NSCLC) and hepatocellular carcinoma cells 191,192, indicating the significance and potential of ACC in both CSCs inhibition and cancer therapy. Similarly, FASN plays an essential part in CSCs' survival and proliferation. Both pharmacological inhibitor and RNA silencing of FASN diminished a variety of CSCs through different disruptive activities, including the destruction of mammosphere formation, weakened invasion, inhibition of proliferation and apoptosis 58,193-195. Currently, FASN inhibitor, such as **TVB-2640**, is in the phase II trials as the assistant drug for chemotherapy (**Paclitaxel**, and **Trastuzumab**) in patients with human epidermal growth factor receptor 2 (HER2) positive advanced breast cancer. Meanwhile, a phase I trial of TVB-2640 is also in patients with colon or other cancers (National Cancer Institute, NCI).

However, therapy by targeting the ACLY seems to be tangled in a whack-a-mole effect. Currently, the progress of ACLY inhibition in CSCs has still been stuck *in vitro* since the year 2013 64. The most controversial issue haunting in the progress is the compensation effect after ACLY inhibition. For example, inhibition or knockdown of ACLY undoubtedly inhibits the growth of certain cancers, but other key players in the fatty acid and cholesterol synthesis pathways, such as FASN and HMGCR, are stimulated in accordingly to reimburse for the effects of ACLY deficiency 55. Furthermore, the duty of ACLY in lipogenesis that converses acetate into acetyl CoA can also be substituted by acetyl-CoA synthetase short-chain family member 2 (ACSS2) in mammals 196. ACSS2 is particularly prominent in the absence of ACLY 60. Therefore, previous studies have speculated that ACSS2 supplements the acetyl CoA required by cells to restore the effects of ACYL inactivation 197, 198. Additionally, ACSS2 also maintains cancer growth under lipid deficiency, and ACSS2 knockdown inhibits tumor xenografts in *vivo* 199. Of note, phosphorylation of ACLY can be conducted by other kinases such as nucleoside diphosphate kinase 200 and cyclic AMP-dependent protein kinase 201. Therapeutic strategies focusing on ACLY phosphorylation also encounters an obstacle, because dephosphorylation and inactivation of ACLY with PI3K inhibitors have no significant effect on lung cancer cell therapy. Though the compensatory effect may not completely rescue the consequences under the absence of ACLY *in vitro* 64, 202, there is still a gap in the effect of ACLY deficiency in cancer research.

### Targets on FAs desaturation

A strong relevance between SCD1 and CSCs suggests a promising therapeutic target for identification and elimination of CSCs. Previous studies aiming at the importance of unsaturated lipids in CSCs also show that SCD1 inhibition by chemical compounds such as **CAY10566**, **A939572**, effectively interferes with cancer stemness, tumor formation and proliferation 35, 90. However, it remains unclear what a consequence on blockage of systemic metabolism would be in normal cells. Ben-David et al showed that an SCD1 inhibitor **PluriSIn-1** effectively eliminates hPSCs while it reserves a sufficient amount of progenitor and differentiated cells 86. Another SCD1 inhibitor **CVT-11127** induces programmed cell death in lung cancer without impairing the proliferation of normal human fibroblasts 89. The current progress of SCD1 inhibitor for cancer therapy mainly stays at the animal test. Comparatively, a liver-specific SCD1inhibitor** MK-8245** is proven to treat diabetes and dyslipidemia without liver toxicity at Phase II clinical trials 203. Noticeably, cocktail inhibitors targeting both the Wnt and Hippo-YAP signallings effectively suppress triple-negative breast cancer in both mesenchymal and epithelial states 204. This finding shows that alteration of lipid metabolism may be a synergy from both the Wnt and YAP pathways in CSCs, indicating an ideal therapeutic strategy. Cocktail inhibitors may be a better option for treating CSCs. No further reports show that SCD1 inhibitor can selectively affect CSCs by sparing normal somatic cells. ALDH family, which is related to the lipid desaturation, is considered as an ideal marker and target for in clinic application. ALDH inhibitors, such as disulfiram and its derivative, achieve periodic results in the promotion of chemosensitization of lung cancer 205.

### Targets on FAO

The therapeutic targets on FAO mainly focus on inhibitors of the rate-limited enzyme CPT1, which locates on the outer mitochondrial membrane. Typically, CPT1 is associated with the increasing level of FAO in glioblastoma and breast, prostate, ovarian, and lung cancer 206. CPT1 inhibitors, such as **etomoxir**, **ranolazine**, reduce chemoresistance and pluripotency of cancer cells 40,97,105,207. Because JAK/STAT3 interferes with cancer stemness through the regulation of CPT1, a first-in-class STAT3 inhibitor displays strong anti-CSC effects in numerous cancers 179. **Napabucasin (BBI608)** is in phase III clinical trials for metastatic colorectal carcinoma and pancreatic cancer 189. Therefore, the potency of FAO inhibition may also create an effective combination for eliminating cancer stemness.

### Targets on cholesterol metabolisms

In melanoma mice models, inhibiting cholesterol esterification by cholesterol acyltransferase (ACAT) inhibitor **avasimibe** leads to enhanced effector function and proliferation of CD8^+^ instead of CD4^+^ T cells 208. Furthermore, ACAT1-deficient CD8^+^ T cells show better control in melanoma growth and metastasis in mice models. In preclinical research, the combination of ACAT inhibitor and an anti-PD-1 antibody exhibits more promising anti-tumor efficiency than monotherapies.

Besides, the activation of LXRs in cancer cells can be induced by disrupting cholesterol metabolism, which regulate inflammation and innate and acquired immunity. In NK cells, activated LXRs leads to overexpression of major histocompatibility complex class I chain-related molecule A and B (MICA and MICB), ligands in melanoma cells rendering the tumor cells more sensitive to recognition, degranulation, and killing by NK cells 209. Cholestane-3β, 5α, 6β-triol (abbreviated as triol) is one of the most abundant and active oxysterols. Triol exhibits anti-cancer activity against human prostate cancer cells. Triol treatment results in reduced expression of Akt1, phospho-Akt Ser473, phospho-Akt Thr308, PDK1, c-Myc 134, and Skp2 as well as accumulation of the cell cycle inhibitor p27^kip^^210^. In a phase II study,** atorvastatin**, a lipophilic statin, is found to prolong survival in HMGCR-positive breast cancer. These observations suggest a novel immune-mediated mechanism involving modulation of intracellular cholesterol levels in cancer cells.

## Summary and Perspectives

Due to the excessive demand for energy and structural component than 'normal' cancer cells, cancer stem cells urgently rely on lipid metabolism to maintain cell survival and proliferation. Dramatically, the known cancer stemness associated signalling pathways, such as **Notch**, **Hippo**, **Wnt**, and **Hh**, have a close relationship with lipid metabolisms. Therefore, the alternation of 'alternated' lipid metabolisms has been indicated as promising therapeutic targets for CSCs suppression and cancer therapy. Specifically, because of the relatively simple and maneuverability for those key regulators in the pathway, therapeutic targets on fatty acid and cholesterol metabolism contribute to several impressive progress on the inhibition of CSCs and reduction of chemoresistance both* in vivo* and *in vitro*.

However, the application also encounters several challenges when commencing in the clinical trials. For example, fatty acid synthesis is drastically upregulated under pathogen infection, a wide range of diseases including cardiovascular disease, insulin resistance of type 2 diabetes and cancers mentioned above 211,212. Hence, the co-occurring of FASN and cancer stemness markers, such as SOX2, CD133 and Nestin, would be hard to hallmark cancer stem cells within multiple pathogenic or metabolic disorder circumstances, especially in tissue with excessive metabolic activities. Secondly, the compensation conversed by other metabolic pathways or uptake from extracellular environment spare cancer cells from the shortage of energy and intermediates for metabolisms. The last but not least, the dilemma crushing on the cancer therapy remains to be the same problem for current treatment; i.e., lipid metabolism-associated inhibitors may also affect surrounding healthy cells, resulting in inevitable side-effects.

Notably, there are several interesting related progresses for therapeutic targets on lipid metabolisms, solving those concerned or problems haunting in current cancer therapy. For instance, the combination of lipid metabolism-associated inhibitors and chemotherapy agents, or immunotherapy (such as PD-1 antibodies), does significantly promote anticancer efficiency. Interestingly, the current study shows another novel strategy by utilizing engineered adipocytes or lipids to deliver the anticancer drug 213,214. By intratumoral or postsurgical injection, this drug design and deliver strategy enable those 'greedy' cancer cells to suffer their consequences. As the global profiles of lipid metabolisms have been well unveiled in cancer or CSCs, we may continuously exploit the combination of exciting therapeutic strategy or a novel treatment, and lipid-associated drugs to ameliorate chemoresistance and even the cure for cancers.

## Figures and Tables

**Figure 4:**
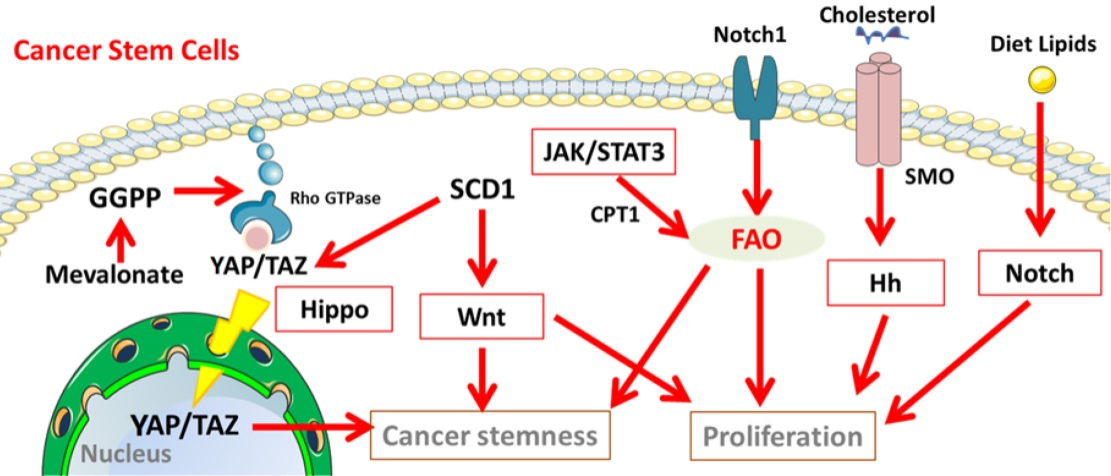
** Cancer stemness related signalling pathways involved in the lipid metabolisms in CSCs.** Notch, Hippo, Hh, and Wnt signalling participate in lipid metabolism to maintain the properties of cancer stem cells. Abbreviation: GGPP, geranylgeranyl pyrophosphate; YAP/TAZ, yes-associated protein (YAP)/ tafazzin (TAZ); JAK/STAT3, Janus kinase/signal transducers and activators of transcription 3; SMO, Smoothened; Hh, Hedgehog. The figure was produced using Servier Medical Art (http://www.servier.com).
